# Prediction of brain age and cognitive age: Quantifying brain and cognitive maintenance in aging

**DOI:** 10.1002/hbm.25316

**Published:** 2020-12-14

**Authors:** Melis Anatürk, Tobias Kaufmann, James H. Cole, Sana Suri, Ludovica Griffanti, Enikő Zsoldos, Nicola Filippini, Archana Singh‐Manoux, Mika Kivimäki, Lars T. Westlye, Klaus P. Ebmeier, Ann‐Marie G. de Lange

**Affiliations:** ^1^ Department of Psychiatry University of Oxford Oxford UK; ^2^ Wellcome Centre for Integrative Neuroimaging University of Oxford Oxford UK; ^3^ NORMENT, Institute of Clinical Medicine University of Oslo, & Division of Mental Health and Addiction, Oslo University Hospital Oslo Norway; ^4^ Centre for Medical Image Computing, Department of Computer Science University College London London UK; ^5^ Dementia Research Centre, Institute of Neurology University College London London UK; ^6^ Epidemiology of Ageing and Neurodegenerative diseases Université de Paris, INSERM U1153 Paris France; ^7^ Department of Epidemiology and Public Health University College London London UK; ^8^ Department of Psychology University of Oslo Oslo Norway

**Keywords:** aging, brain maintenance, cognitive reserve, lifestyle, machine learning, neuroimaging, trajectories

## Abstract

The concept of *brain maintenance* refers to the preservation of brain integrity in older age, while *cognitive reserve* refers to the capacity to maintain cognition in the presence of neurodegeneration or aging‐related brain changes. While both mechanisms are thought to contribute to individual differences in cognitive function among older adults, there is currently no “gold standard” for measuring these constructs. Using machine‐learning methods, we estimated brain and cognitive age based on deviations from normative aging patterns in the Whitehall II MRI substudy cohort (*N* = 537, age range = 60.34–82.76), and tested the degree of correspondence between these constructs, as well as their associations with premorbid IQ, education, and lifestyle trajectories. In line with established literature highlighting IQ as a proxy for cognitive reserve, higher premorbid IQ was linked to lower cognitive age independent of brain age. No strong evidence was found for associations between brain or cognitive age and lifestyle trajectories from midlife to late life based on latent class growth analyses. However, post hoc analyses revealed a relationship between cumulative lifestyle measures and brain age independent of cognitive age. In conclusion, we present a novel approach to characterizing brain and cognitive maintenance in aging, which may be useful for future studies seeking to identify factors that contribute to brain preservation and cognitive reserve mechanisms in older age.

## Graphical abstract

Using machine learning, we estimated brain and cognitive age based on deviations from normative aging patterns in the Whitehall II MRI substudy cohort, and tested the degree of correspondence between these constructs, as well as their associations with premorbid IQ, education, and lifestyle trajectories. The study presents a novel approach to characterizing brain and cognitive maintenance in aging, which may be useful for future studies seeking to identify factors that contribute to brain preservation and cognitive reserve in older age.

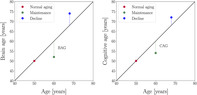

## 1 INTRODUCTION

Most cognitive abilities are well established to decline with age (Grady, 2012), and cognitive deterioration can to some extent be attributed to concurrent changes in brain structure (Bennett & Madden, 2014; Fjell et al., 2016; Fjell, Sneve, Grydeland, Storsve, & Walhovd, 2017; Fjell & Walhovd, 2010; Nyberg, Dahlin, Stigsdotter Neely, & Bäckman, 2009). Age‐related structural changes typically manifest as reductions in brain volume, cortical thinning, and decline in white matter (WM) microstructure (Fjell et al., 2014), which can lead to poorer cognitive performance in domains such as executive function, memory, and processing speed (Cabeza et al., 2018; Grady, 2012; Nyberg, Lövdén, Riklund, Lindenberger, & Bäckman, 2012). However, the aging population is characterized by considerable variation between individuals, and while some develop cognitive impairment, Alzheimer's disease, and other types of dementia, others may to a large extent maintain their cognitive function well into late life (Nyberg et al., 2012).

### 1.1 Brain maintenance and cognitive reserve

Interindividual differences within older adult populations have led to a large number of studies focusing on risk and protective factors for cognitive decline in aging (Anatürk, Demnitz, Ebmeier, & Sexton, 2018; Bråthen, Lange, Fjell, & Walhovd, 2020; Nyberg, Fjell, & Walhovd, 2019; Sabia et al., 2019; Zsoldos et al., 2018), as well as factors that characterize successful aging or “SuperAgers” (Gefen et al., 2014; Harrison, Weintraub, Mesulam, & Rogalski, 2012; Rogalski et al., 2013; Yu et al., 2019). The maintenance of a “younger” brain, that is, the relative lack of aging‐related changes including pathology, has been suggested as a main mechanism to preserve cognitive function into older age (Nyberg et al., 2012). For example, while decline in cortical thickness is commonly observed between midlife and late life, a unique group of older adults (i.e., “SuperAgers”) do not exhibit this typical pattern of cortical atrophy. SuperAgers also appear to possess higher cortical thickness in some brain regions relative to younger individuals (Rogalski et al., 2013), and perform comparably to young adults on assessments of memory (Harrison et al., 2012; Sun et al., 2016). However, a number of clinical studies have reported weak associations between degree of brain pathology and relevant cognitive symptoms (Scarmeas & Stern, 2003), and as a consequence, *reserve theories* have gained prominence in the aging field (Driscoll et al., 2006; Snowdon, 2003; Stern, 2002; Stern, 2009).

The *cognitive reserve theory* seeks to explain why some individuals are able to maintain cognitive function in the presence of pathology or aging‐related brain changes. It has been suggested that individuals with higher cognitive reserve process information more efficiently, enabling them to functionally adapt to brain aging and sustain greater pathology before cognitive impairments manifest (Stern, 2002). Education has been suggested to promote cognitive reserve by enhancing cognitive flexibility, and factors such as lifestyle behaviors may moderate the beneficial effect of education on cognition in older age (Cabeza et al., 2018). As there is no established method to directly measure cognitive reserve, the majority of reserve studies have used measures such as IQ or education as proxies of reserve (Valenzuela & Sachdev, 2006a; Valenzuela & Sachdev, 2006b), and focused on how cognitive function and brain metrics differ between individuals with high or low levels of this proxy measure (Cabeza et al., 2018).

In summary, preservation of brain structure and cognitive reserve mechanisms can both potentially contribute to a higher degree of maintained cognitive function in older age. Brain maintenance and cognitive reserve can thus be viewed as complementary constructs that may be malleable over the lifespan (Cabeza et al., 2018), potentially serving as targets for lifestyle interventions. However, despite their centrality to studies of aging (Stern, 2003), there remains no consistent approach to measuring and comparing these constructs. In this article, we refer to maintained brain structure and cognitive function relative to normative age trajectories as *brain maintenance* and *cognitive maintenance*. A strong association between the two would imply correspondence between degrees of brain and cognitive maintenance, while a weak association could reflect a lack of one‐to‐one relationships between these trajectories—potentially providing evidence for *cognitive reserve* mechanisms.

**Table:** 

**Definition of key concepts**
*Brain maintenance*
Preserved brain structure in older age relative to normative age trajectories
*Cognitive maintenance*
Preserved cognitive function in older age relative to normative age trajectories
*Cognitive reserve*
Preserved cognitive function in the presence of aging‐related brain changes

### 1.2 Prediction of brain age and cognitive age

**FIGURE 1:**
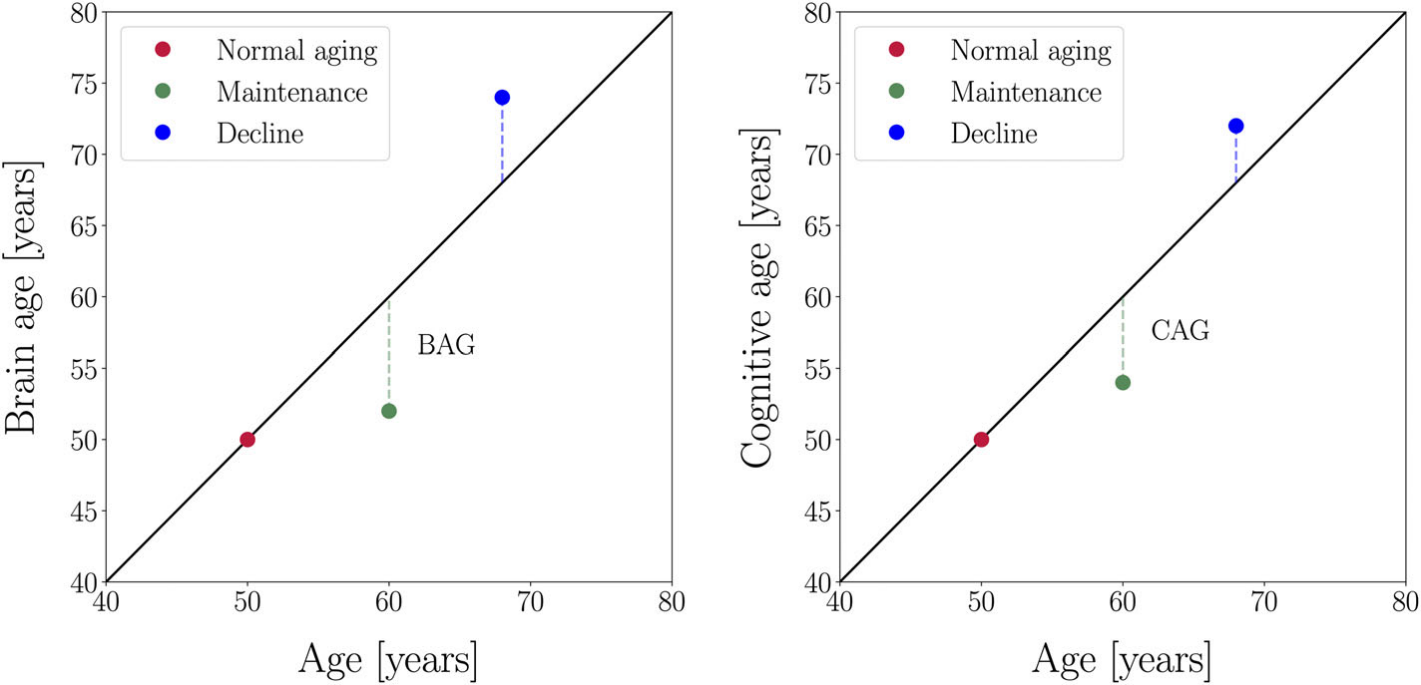
Conceptual illustration of brain age (left) and cognitive age (right), where the distance between estimated brain age/cognitive age (colored dots) and the expected brain age/cognitive age (black line) represents brain age gap (BAG) and cognitive age gap (CAG)

The application of machine learning to neuroimaging data has provided an avenue for estimating the apparent age of an individual's brain, and determining deviations from normative brain aging patterns (Cole, Marioni, Harris, & Deary, 2019). Studies in this area suggest that the difference between estimated “brain age” and chronological age (i.e., brain age gap [BAG]) varies between individuals, with positive BAG values (older brain age relative to chronological age) relating to poorer cognitive function (Boyle et al., 2020; Cole et al., 2018; Elliott et al., 2019). A recent multicohort study of 45,615 individuals further highlighted that BAG may be a sensitive marker of disease, with accelerated brain aging observed in a range of conditions including mild cognitive impairments, Alzheimer's disease, and depression (Kaufmann et al., 2019). Mapping this metric onto the concepts introduced in Section 1.1, BAG estimated from structural MRI may reflect degree of *brain maintenance* (Steffener et al., 2016), with negative values suggesting preserved brain structure relative to what is expected based on normative trajectories (as illustrated in Figure 1). Based on this rationale, machine learning algorithms can also be applied to cognitive data to provide an estimate of cognitive age, with the difference between cognitive and chronological age (i.e., cognitive age gap [CAG]) reflecting degree of *cognitive maintenance* (see Figure 1). The correlation between BAG and CAG would indicate the degree of correspondence between brain and cognitive maintenance or decline relative to normative aging patterns, as illustrated in Figure 1. Developing methods for characterizing brain and cognitive maintenance is timely, as there is a growing demand for lifestyle‐based interventions that may help prevent or delay age‐ and disease‐related cognitive decline (Livingston et al., 2017).

### 1.3 Lifestyle predictors of cognitive/neural integrity

The extent to which BAG and CAG relate to known predictors of healthy aging may shed light on the associations between lifestyle factors and maintenance/reserve mechanisms. For example, educational level may relate to cognitive maintenance independent of brain maintenance, potentially suggesting that education facilitates preserved cognition via cognitive reserve mechanisms. Health behaviors such as abstaining from smoking (Zhong, Wang, Zhang, Guo, & Zhao, 2015), limiting the amount of alcohol consumed (Rehm, Hasan, Black, Shield, & Schwarzinger, 2019), and achieving a sufficient amount of physical activity (PA; Guure, Ibrahim, Adam, & Said, 2017) could promote maintained cognitive function (Cadar et al., 2012; Clare et al., 2017; Sabia et al., 2009) and decreased dementia risk (Lourida et al., 2019) through brain preservation, as indicated by studies showing a link between these factors and estimates of BAG (Cole, 2020; de Lange, Anatürk, et al., 2020; Smith et al., 2020; Steffener et al., 2016). However, a substantial proportion of studies on lifestyle factors and brain/cognitive aging are based on cross‐sectional measurements (Anatürk et al., 2018; Scarmeas & Stern, 2003) or on self‐reported history, which is highly susceptible to recall biases (Gow, Pattie, & Deary, 2017). As *prolonged* or *cumulative* exposure to a healthy lifestyle has been suggested to play an important role in individual variation in health outcomes (Ben‐Shlomo, Cooper, & Kuh, 2016; Whalley, Dick, & McNeill, 2006), a lifespan perspective may be critical for understanding how lifestyle factors relate to cognitive/neural aging and dementia onset. For example, lower average alcohol consumption over 30 years is linked to higher regional gray matter (GM) density and WM integrity in older adults (Topiwala et al., 2017). While examining cumulative exposure is a useful approach, it provides limited insight into the specific *lifestyle trajectories* conducive to cognitive and brain aging. Recent data‐driven approaches have begun to characterize the different lifestyle patterns that co‐occur between midlife and late life. For instance, a coordinated analysis of data from six cohorts (based in the United States, England, Europe, Japan, Korea, and China) suggested that there may be three major “clusters” of middle‐aged and older adults, including (a) those who engaged in multiple healthy behaviors, (b) those who were socially and physically inactive but did not engage in risky behaviors, and (c) ex‐smokers engaging in other risk behaviors (Liao et al., 2019). Other studies report between two and nine subgroups (reviewed in Noble, Paul, Turon, and Oldmeadow (2015)). The majority of this evidence, however, is based on cross‐sectional survey data, and the ways in which brain preservation or cognitive reserve mechanisms may serve as potential pathways between lifestyle trajectories and late‐life cognition are not well understood.

### 1.4 Study aims

In the current study, we used machine learning models to estimate (a) brain age based on MRI‐derived measures, and (b) cognitive age based on performance scores on several cognitive tests. For each participant, we calculated an estimate of BAG (predicted brain age minus chronological age, indicating degree of *brain maintenance* (Steffener et al., 2016)), and CAG (predicted cognitive age minus chronological age, i.e., degree of *cognitive maintenance*). We first correlated BAG with CAG to investigate the degree of correspondence between brain and cognitive maintenance. Second, we tested the associations of CAG and BAG with premorbid IQ and education, which are known as proxies of cognitive reserve (Cabeza et al., 2018). Finally, we employed a latent class growth analysis (LCGA) to characterize lifestyle trajectory classes based on five repeated measures of lifestyle behaviors (PA, smoking status, and alcohol intake) between midlife to late life, and tested the associations between trajectory class and BAG/CAG. In addition, we calculated an index of cumulative lifestyle behaviors based on a composite score across measurement timepoints, and tested the association of this index with BAG and CAG.

## 2 MATERIALS AND METHODS

### 2.1 Sample

Data from participants enrolled in the Whitehall II imaging substudy were examined. A detailed description of this cohort has been published previously (Filippini et al., 2014). In brief, these individuals were originally recruited in 1985 as part of a cohort of 10,308 civil servants based in London (Marmot & Brunner, 2005), and have since been regularly assessed (at ∼5 year intervals) on a range of lifestyle, biological, and cognitive variables. Between 2012 and 2016, a random sample of 800 individuals were invited to undergo an MRI brain scan and comprehensive cognitive battery, as part of the Whitehall II imaging substudy (Filippini et al., 2014).

**FIGURE 2:**
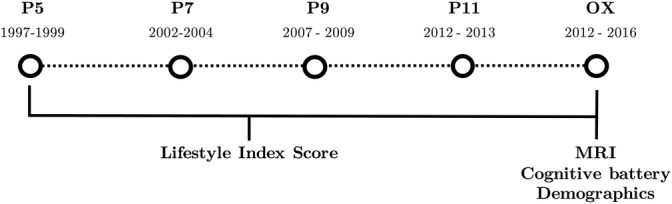
An overview of the variables of interest provided at each phase of the WHII study (for a full description of data available at all study phases, please see https://www.ucl.ac.uk/epidemiology‐health‐care/research/epidemiology‐and‐public‐health/research/whitehall‐ii/data‐collection). Phases were selected based on the availability of measures of alcohol consumption, smoking habits, and physical activity. Composite measures of healthy lifestyle scores were derived from five phases (average length of time [mean ± *SD*] = 16.3 years ± 1.4), with an MRI scan administered at the fifth timepoint

The current sample was drawn from the Imaging Sub‐study cohort, and included 537 participants who had provided lifestyle information for at least four previous study phases. Additional criteria for inclusion were complete MRI, cognitive and demographic data at the MRI phase, no artifacts or substantial motion in the MRI images, no structural abnormalities detected in the MRI scans (e.g., strokes or tumors) and no self‐report neurological diagnoses (e.g., Parkinson's disease) or a current SCID diagnosis of depression, anxiety, psychosis, or cognitive disorder. A description of sample characteristics is provided in Table 1, and an overview of the study timeline is available in Figure 2. The study received ethical approval from the University of Oxford Central University Research Ethics Committee, as well as the University College London Medical School Committee on the Ethics of Human Research. All participants enrolled in this study gave their informed and written consent.

**TABLE 1:** Sample characteristics N = 537

Variables	Mean ± *SD*	Range
Age at MRI scan (years)	69.75 ± 5.08	60.34–82.76
Sex		
Female (%)	94 (17.50%)	
Education (years)	16.75 ± 4.44	7–44
Ethnicity		
White (%)	508 (94.60%)	
MoCA (score)	27.31 ± 2.16	18–30
Healthy lifestyle score^a^		
Phase 5	1.11 ± 0.85	0–3
Phase 7	1.16 ± 0.84	0–3
Phase 9	1.24 ± 0.84	0–3
Phase 11	1.27 ± 0.87	0–3
Phase OX	2.05 ± 0.66	0–3
Cumulative lifestyle score	5.07 ± 2.96	0–12.6

Abbreviation: MoCP, Montreal cognitive assessment.

^a^ Scale is number of self‐reported health behaviors. While raw values are reported here, these variables were standardized for the analyses.

### 2.2 Demographics

All demographic variables were assessed at the time of scan. Education was measured as total years of full time and part time education. Ethnicity was defined as White/Non‐white. Body mass index (BMI) was calculated using information on height and weight: weight [kg]/(height [m])^2^.

### 2.3 MRI data acquisition and processing

The participants underwent a 3 T MRI scan at the Centre for Functional Magnetic Resonance Imaging of the Brain, Wellcome Centre for Integrative Neuroimaging, at the University of Oxford. Between April 2012 and December 2014, scanning was conducted with a Siemens Magnetom Verio with a 32‐channel receive head coil (No. participants = 390). Following a scanner upgrade in early 2015, further scanning was conducted on a Siemens Magnetom Prisma with a 64‐channel head–neck coil (No. participants = 147). T1‐weighted (GM volume), diffusion‐weighted (WM microstructure), and fluid attenuated inversion recovery (FLAIR, WM lesions) images were examined in this study. For details of the MRI acquisition parameters for each modality, please see Filippini et al. (2014) and Zsoldos et al. (2018).

#### 2.3.1 MRI features

The GM feature preparation followed the procedures described in previous studies (de Lange et al., 2019; de Lange, Anatürk, et al., 2020; Kaufmann et al., 2019). Briefly, the features were derived using a fine‐grained cortical parcellation scheme (Glasser et al., 2016) including global and region‐specific measures of surface area, volume, and cortical thickness, in addition to the classic set of subcortical volume parcellations and summary statistics based on the automatic segmentation in FreeSurfer (Fischl et al., 2002). The GM variables were residualized with respect to scanner, relative head motion measured during the acquisition of resting state fMRI images (in line with previous studies that have adjusted for motion‐related variance in structural measures (Miller et al., 2016; Smith et al., 2015)), sex, ethnic background, and intracranial volume (ICV (Voevodskaya et al., 2014)) using linear models. As a crosscheck, we ran an additional brain‐age model using cortical thickness features that were left out of the general ICV‐correction.

WM features including global and tract‐specific estimates of fractional anisotropy (FA), mean diffusivity (MD), axial diffusivity (AD), radial diffusivity (RD), and mode of anisotropy (MO) were derived using 48 standard‐space masks available from the ICBM‐DTI‐81 White‐Matter Labels Atlas (Mori, Wakana, van Zijl, & Nagae‐Poetscher, 2005; Wakana et al., 2007). Prior to this step, the diffusion‐weighted images were preprocessed using a combination of FSL's tools, including *Top Up* to correct for eddy currents and head motion, DTIFIT to fit the diffusion tensor and extract images for each metric of interest (e.g., FA and RD images), which was followed by tract‐based spatial statistics for registration and extracting FA/MD/AD/RD/MO skeletons for each individual (for further details, see supplementary methods in Anatürk et al. (2020). Global WM hyperintensity (WMH) volumes were automatically extracted from FLAIR images with Brain Intensity AbNormality Classification Algorithm, as described in Griffanti et al. (2016). To test for improvements in model performance using more detailed measures of WMH, we also included an extended model with separate measures for deep and periventricular WMH (Armstrong et al., 2020; Griffanti et al., 2018). The WM variables were residualized with respect to scanner, relative head motion, sex, and ethnic background using linear models.

A total of 1,118 GM features and 245 WM features were included in the brain age prediction model. For comparison purposes, a reduced model was run using one measure per modality as input variables (total GM volume, average FA, and total WMH volume).

### 2.4 Cognitive features

A cognitive battery was administered on the day of the MRI scan by either a trained research assistant or psychiatrist. The tests included measures of premorbid IQ (test of premorbid functioning; Wechsler, 2011), global cognition (Montreal cognitive assessment [MoCA]; (Nasreddine et al., 2005)), working memory (Digit Span: forward, backward, ascending sequence (Wechsler, 2008)), visual attention and task‐switching (Trail Making Test; TMT A & B; (Reitan, 1958)), visuospatial memory (Rey–Osterrieth complex figure: immediate and delayed recall, recognition (Osterrieth, 1944)), verbal memory (Hopkins Verbal Learning Test: immediate and delayed recall, recognition (Brandt, 1991)), semantic memory (Boston Naming Test (Kaplan, Goodglass, & Weintraub, 2001)); verbal fluency (*adapted from the Addenbrookes Cognitive Examination Revised* (Hsieh, Schubert, Hoon, Mioshi, & Hodges, 2013): semantic and letter fluency); processing speed (Digit Coding (Wechsler, 2008)), and simple and complex reaction time (CANTAB RTI; CANTABeclipse 5.0; Cambridge Cognition Ltd. Measures: simple reaction time; choice reaction time; simple movement time; and choice movement time). For a detailed description of these tests, please see Filippini et al. (2014).

### 2.5 Lifestyle index scores

Lifestyle index scores were calculated separately for each of the five study phases shown in Figure 2. The index was derived based on an individual's position on three behavioral variables: alcohol intake, smoking status, and PA. In general, participants received a point of 1 for each behavior if they met governmental guidelines and/or recommendations outlined in the literature, with the lifestyle index scores ranging from 0 (reporting no healthy behaviors) to 3 (reporting three healthy behaviors).

For alcohol consumption, the measurement was based on self‐reported total units consumed on a weekly basis (over the last year). Individuals who self‐reported as abstinent from alcohol (<1 unit/week) or engaged in light drinking (i.e., 1–7 units/week) were assigned a point of 1. As a prior analysis of the Whitehall sample (Topiwala et al., 2017) recently suggested that moderate levels of alcohol may be associated with adverse brain outcomes, individuals consuming more than 7 units per week received a score of 0 (classified as “unhealthy”).

For smoker status, individuals were assigned a point of 1 if they had never smoked, whereas individuals who were current or previous smokers were given a score of 0 (as done in prior studies: (Sabia et al., 2009; Zhong et al., 2015)). At the MRI time point, smoker status was inferred from two items of the lifestyle questionnaire,” *Have you smoked cigarettes in the past four weeks?”* and” *How many cigarettes in a typical week?”*

For PA, an individual reporting at least 2.5 hr of moderate to vigorous intensity activities per week (Metabolic equivalent of task values > 3, assignments were coded as 1 based on previous findings (Sabia et al., 2017), with all other individuals scoring 0. The measurement and computation of these variables are described in greater detail in Sabia et al. (2017). At the time of scan, the Community Healthy Activities Model Program for Seniors (CHAMPS (Stewart et al., 2001)) questionnaire was used to measure engagement (frequency and duration) in a range of 41 activities per week, including 20 moderate to vigorous intensity activities (e.g., playing tennis, aerobics exercises, strength training).

### 2.6 Statistical analyses

#### 2.6.1 Brain age and cognitive age

A regression model from *XGBoost* (https://xgboost.readthedocs.io/en/latest) was used to predict brain age based on the MRI measures described in Section 2.3.1, and cognitive age based on the cognitive tests described in Section 2.4 (excluding premorbid IQ). The *XGBoost* model is based on a decision‐tree ensemble algorithm that includes advanced regularization to reduce overfitting (Chen & Guestrin, 2016), and uses a gradient boosting framework where the final model is based on a collection of individual models (https://github.com/dmlc/xgboost). To optimize hyperparameters, a randomized search was performed with 10 folds for each model, with scanned parameter ranges set to *maximum depth*: (Bennett & Madden, 2014; Bråthen et al., 2020; Grady, 2012), *number of estimators*: [60, 220, 40], and *learning rate*: [0.1, 0.01, 0.05]. The optimized parameters were *maximum depth* = 2, *number of estimators* = 180, and *learning rate* = 0.1 for the brain age model, and *maximum depth* = 2, *number of estimators* = 140, and *learning rate* = 0.05 for the cognitive age model. In addition, a grid search with nested cross‐validation was performed to test for potential overfitting. All MRI and cognitive variables were standardized before being entered as features into these analyses.

To assess the significance of the general model performances, average *R*^2^, root mean square error (RMSE), and mean absolute error (MAE) were estimated for each model using cross‐validation with 10 splits and 5 repetitions, and compared to null distributions calculated from 1,000 permutations. The 10‐fold cross‐validations produced an estimate of each measure for every individual in the sample. To investigate the prediction accuracy, correlation analyses were run for predicted brain age versus chronological age and predicted cognitive age versus chronological age. *R* ^2^, RMSE, and MAE were calculated for each model using the Scikit‐learn library with Python (version 3.7.0). An overview of how BAG and CAG estimates were calculated is available in Table 2. To investigate the associations between BAG and CAG, we first corrected the estimations for chronological age using linear regression (Le et al., 2018) before correlating the BAG and CAG residuals.

**TABLE 2:** A summary of the main dependent variables of the study

Variable	Calculation	Negative values=	Concept
BAG	(Brain age—chron. age)	Younger brain age relative to chron. age	Brain maintenance	
CAG	(Cognitive age—chron. age)	Younger cognitive age relative to chron. age	Cognitive maintenance	

Abbreviations: BAG, brain age gap; CAG, cognitive age gap.

#### 2.6.2 Associations with education and premorbid IQ

Linear regressions were performed to evaluate whether CAG and BAG associated with education or premorbid IQ, which are known predictors of cognitive reserve (Kartschmit, Mikolajczyk, Schubert, & Lacruz, 2019). These associations were first adjusted for age only, and if an association was found, sex, education, ethnicity, BMI, as well as CAG/BAG were included as covariates in a follow‐up analysis to adjust for potential confounders. If education or premorbid IQ were significantly associated with either BAG or CAG (at an alpha [*α*] level of .05), we directly compared these covariate adjusted associations using Z‐tests for correlated samples (Zimmerman, 2012) with:

(1) $$Z=\left(\beta_{m1}-\beta_{m2}\right)/\sqrt{\sigma_{m1}^{2}+\sigma_{m2}^{2}-2{\rho \sigma }_{m1}\sigma_{m2}},$$

where m1 = Model 1 (CAG); m2 = Model 2 (BAG); *β* = beta coefficients from the covariate adjusted linear regressions between variables of interest and BAG/CAG; *σ* = *SE*s of the beta coefficients; and *ρ* = age‐adjusted correlation between BAG and CAG.

#### 2.6.3 Associations with lifestyle trajectories

LCGA was employed to evaluate whether the sample could be sub‐divided into trajectory classes based on repeated measures of lifestyle index scores (Section 2.5). This is a widely used method that has previously been applied to identify trajectories of sleep (Zitser et al., 2020) and BMI (Vistisen et al., 2014) in the Whitehall II cohort. LCGA was run with the following specifications: *No*. *random sets of starting values*: 500, *optimizations*: 20, and *iterations*: 20. *Time metric*: Mean age at each assessment was used as the metric of time, with each time score centered by 53 (mean baseline age) and divided by 10 to aid model convergence: [0, 0.3, 1.1, 1.5, 1.7]. *Estimator*: restricted maximum likelihood with robust *SE*s was used. Full information maximum likelihood was also employed to estimate parameters in the presence of missing data (Enders & Bandalos, 2001). All lifestyle index scores were standardized prior to the LCGA to minimize biases introduced by variation in the questionnaire between the prior phases and MRI scan.

The model with best fit was identified based on achieving the lowest Akaike information criterion (AIC), Bayesian information criterion (BIC), and sample‐size adjusted BIC (aBIC), with an entropy value ≥0.8 and a *p*‐value <.05 for the Vuong–Lo–Mendell–Rubin (VLMR) likelihood ratio and bootstrap likelihood ratio (BLRT) tests (Jung & Wickrama, 2008). A minimum of 5% of participants within each class was also required to minimize the likelihood that a class captured outliers only (Andruff, Carraro, Thompson, Gaudreau, & Louvet, 2009). These analyses were conducted with MPlusAutomation (version 0.7‐3 (Hallquist & Wiley, 2018)).

To test for relationships between lifestyle trajectories and estimates of brain and cognitive maintenance, BAG and CAG were used as dependent variables in separate linear regressions. Lifestyle trajectory classes were entered as independent variables and as dummy coding was used, the class with the least favorable lifestyle served as the reference category. In addition, a cumulative lifestyle score based on an average of lifestyle scores across all phases was examined as a potential predictor of BAG and CAG in separate regression analyses. An average was selected over a summary score due to being less biased in the presence of missing data (as individuals with either four or five phases of lifestyle data were included in the analyses). In order to derive comparable *β* estimates, all variables were standardized prior to the linear regression analyses. The associations were first examined adjusting for chronological age only (Model 1), and if the associations were significantly different from zero (*α* level of .05), they were followed up by analyses adjusting for age as well as CAG/BAG, and additional factors known to influence brain and cognitive health in aging, including sex (e.g., Ritchie et al., 2018), education (e.g., Boller, Mellah, Ducharme‐Laliberté, & Belleville, 2017), ethnicity (e.g., Gavett et al., 2018), and BMI (e.g., Dekkers, Jansen, & Lamb, 2019), (Model 2). If the relationships remained significant (prior to multiple comparisons), the associations were directly compared using the z‐test for correlated samples as described in Equation (1).

#### 2.6.4 Multiple comparison corrections and sensitivity analyses

All hypothesis tests were two‐sided. We report unadjusted *p*‐value (*p*) and *p*‐values corrected for multiple comparisons using false discovery rate (FDR) correction (Benjamini & Hochberg, 1995) (*p*_*corr*_). As linear regression and z‐tests for correlated samples were performed multiple times, we report the associations that showed a *p*‐value <.05 but did not survive FDR‐correction as *trends*, and associations that showed *p*_*corr*_ <.05 as *significant*. The linear regression analyses and Z‐tests for correlated samples were rerun after outliers on any of the variables of interest were excluded (i.e., individuals with values <Q1–3 × interquartile range [IQR] or values >Q3 + 3 × IQR).

## 3 RESULTS

### 3.1 Brain age and cognitive age

Average *R*^2^, RMSE, and MAE for the brain age and cognitive age models are provided in Table 3. Supplementary Information (SI) Figure 1 shows the average *R*^2^, RMSE, and MAE for each of the models compared to null distributions. The number of permuted results from the null distribution that exceeded the mean from the cross‐validation was 0 for both the brain age and cognitive age models (*p* = 1.00 × 10^−3^).

**TABLE 3:** Average *R* ^2^, RMSE, and MAE ± *SD*s for each of the models, and correlations (*r*) between predicted brain/cognitive age and chronological age. 95% confidence intervals are indicated in square brackets. RMSE and MAE are reported in years

Model	*R* ^2^	RMSE	MAE	*r* [95% CI]	*p*
Brain age	.38 ± .11	3.86 ± 0.36	3.11 ± 0.33	.63 [0.58, 0.68]	<.001
Cognitive age	.09 ± .10	4.70 ± 0.45	3.84 ± 0.39	.31 [0.23, 0.38]	<.001

Abbreviations: MAE, mean absolute error; RMSE, root mean square error; *SD*, standard deviation.

The brain age model showed a higher correlation between predicted and chronological age (*r* = .63; 95%CI [0.58, 0.68]) compared to the cognitive age model (*r* = .31; 95% CI [0.23, 0.38]), as shown in Table 3 and SI Figure 2. To test whether the relative difference in prediction accuracy depended on number of features included, the models were rerun using principal component analysis (PCA) transformed variables with the top 20 components as input for each model. The relative accuracy of the two models was consistent with the main results, as showed in Table 4.

**TABLE 4:** Average *R* ^2^, RMSE, and MAE ± *SD*s using the top 20 PCA components as input for each of the models, explaining 43.63% of the variance in the MRI data, and 100% of the variance in the cognitive data. 95% confidence intervals are indicated in square brackets. RMSE and MAE are reported in years

Model	*R* ^2^	RMSE	MAE	*r* [95% CI]	*p*
Brain age	.28 ± .10	4.15 ± 0.35	3.32 ± 0.31	.55 [0.49, 0.61]	<.001
Cognitive age	.07 ± .10	4.73 ± 0.41	3.86 ± 0.37	.33 [0.25, 0.40]	<.001

Abbreviations: MAE, mean absolute error; PCA, principal component analysis; RMSE, root mean square error; *SD*, standard deviation.

The results from the nested cross‐validation with grid search were similar to the main results, as shown in SI Table S1. For the brain‐age model, leaving cortical thickness out of the general GM ICV‐correction did not change the results, as shown in SI Tables S2 and S3. While the extended model with separate measures for deep and periventricular WMH did not improve the model prediction, the reduced model using one summary measure per modality as input showed lower prediction accuracy, as shown in SI Table S4.

Predicted brain age correlated positively with predicted cognitive age (Pearson's *r* = .24, 95% CI = [0.16, 0.31], *p* < .001), indicating a moderate correspondence between cross‐sectional estimates of normative brain and cognitive age trajectories. The age‐adjusted association between BAG and CAG was not significant (Pearson's *r* = .06, 95% CI = [−0.03, 0.14], *p* = .17). As a crosscheck, we tested whether BAG was associated with global and domain‐specific cognition (Boyle et al., 2020; Cole et al., 2018; Elliott et al., 2019). A subset of tests were used to define domains of executive function, memory and processing speed, in line with prior studies (Demnitz et al., 2017; Sexton et al., 2017). The results showed that BAG did not correlate with measures of global (MoCA scores) or domain‐specific cognitive function, as shown in SI Table S5.

### 3.2 Associations with education and premorbid IQ

The CAG/BAG associations with education and premorbid IQ are provided in Table 5. Both higher premorbid IQ and years of education related to a younger cognitive age relative to chronological age (*β* = −.08, 95% CI = [−0.11, −0.04], *p* < .001, *p*_*corr*_ < .001; *β* = −.06 95% CI = [−0.09, −0.02], *p* = .001, *p*_*corr*_ = .005, respectively). After covariate adjustments, the association between premorbid IQ and CAG survived, while the association between education and CAG was nonsignificant. No associations were found between BAG and premorbid IQ or education, as shown in Table 5. Z‐tests for correlated samples (using Equation (1)) showed that premorbid IQ related more strongly to CAG relative to BAG (*Z* = −3.13, *p* < .001, *p*_*corr*_ = .472) as shown in Table 6.

**TABLE 5:** Associations between CAG/BAG estimates and education, premorbid IQ, and cumulative healthy lifestyle scores. 95% confidence intervals are indicated in square brackets. Model 1 was adjusted for chronological age only. Associations that were significant (before multiple comparison corrections) were submitted to linear regression with additional covariates (i.e., Model 2). Model 2 was adjusted for age, sex, education, ethnicity, BMI, and mutual adjustments between BAG and CAG. Note, where education and premorbid IQ were the independent variables of interest, mutual adjustments were also included in Model 2 (with BMI excluded from the list of covariates). Confidence intervals are indicated in square brackets. *P*‐values are provided before and after FDR‐correction.

		Model 1			Model 2		
DV	IV	*β* [95% CI]	*p*	*p*_*corr*_	*β* [95% CI]	*p*	*p*_*corr*_
CAG	Education	−.06 [−0.09, −0.02]	.001	.005	−.01 [−0.06, 0.03]	.559	.559
	Premorbid IQ	−.08 [−0.11, −0.04]	<.001	<.001	−.07 [−0.12, −0.02]	.004	.015
	Cumulative lifestyle	−.01 [−0.05, 0.02]	.473	.526	—	—	
	Lifestyle: Mod vs. low	−.01 [−0.10, 0.07]	.765	.765	—	—	
	Lifestyle: High vs. low	−.07 [−0.16, 0.02]	.126	.253	—	—	
BAG	Education	−.03, [−0.09, 0.02]	.208	.347	—	—	
	Premorbid IQ	.02 [−0.03, 0.08]	.372	.526	—	—	
	Cumulative lifestyle	−.06 [−0.12, −0.01]	.018	.058	−.06 [−0.12, −0.01]	.029	.064
	Lifestyle: Mod vs. low	−.05 [−0.19, 0.08]	.435	.526	—	—	
	Lifestyle: High vs. low	−.14 [−0.29, −0.00]	.045	.114	−.13 [−0.28, 0.01]	.065	.087

Abbreviations: BAG, brain age gap; BMI, body mass index; CAG, cognitive age gap; DV, dependent variable; FDR, false discovery rate; IV, independent variable.

**TABLE 6:** Difference between the BAG and CAG associations with premorbid IQ and cumulative lifestyles (adjusting for all covariates). For the calculation used to compare the difference between associations, see Equation (1) (Section 2). Confidence intervals are indicated in square brackets. *P*‐values are provided before and after FDR‐correction

Variable	*β*_*CAG*_	*β*_*BAG*_	*Z*	*p*	*p*_*corr*_
Premorbid IQ	−.07 [−0.12, −0.02]	.08 [0.00, 0.16]	−3.24	<.001	.002
Cumulative lifestyle	−.01 [−0.05, 0.02]	−.06 [−0.12, −0.01]	1.398	.162	.162

Abbreviations: BAG, brain age gap; CAG, cognitive age gap; FDR, false discovery rate.

### 3.3 Lifestyle trajectories

**FIGURE 3:**
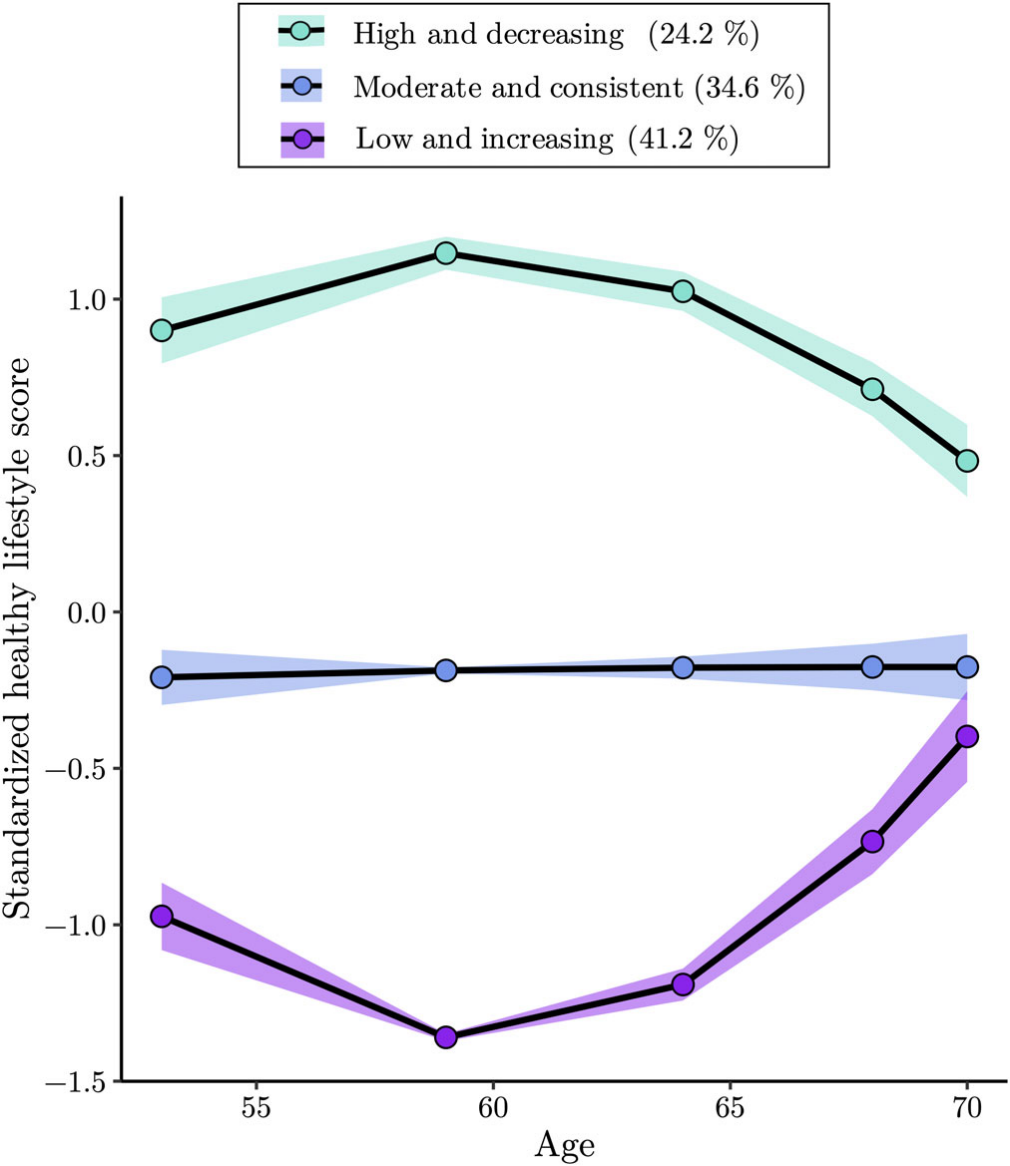
Trajectories of the three‐class solution that was identified as the best fit, which are described in Section 3.3. Shaded areas reflect 95% confidence intervals

The results of the LCGA are provided in SI Table S6 and Figure 3. A three‐class quadratic solution provided the best fit (AIC = 6,098.21, BIC = 6,166.79, aBIC = 6,116.00, BLRT *p* = <.001, VLMR *p* = .04). The class description and percentages per class were: *High but declining lifestyles* (Class 2, *n* = 130, 24.2%), *Moderate and consistent lifestyles* (Class 3, *n* = 186, 34.6%) and *Low but improving lifestyles* (Class 1, *n* = 221, 41.2%).

The CAG/BAG associations with lifestyle trajectories are provided in Table 5. After adjusting for chronological age, a significant association was observed for *high and decreasing* lifestyle trajectories with BAG (*β* = −.14, 95% CI = [−0.29, −0.00], *p* = .045). However, this association did not survive further covariate adjustments and corrections for multiple comparisons (*β* = −.13, 95% CI = [−0.28, −0.01], *p* = .065, *p*_*corr*_ = .087). No associations with BAG were identified for *moderate and consistent* lifestyle trajectories, as compared to *low and increasing* lifestyle (the reference group). No significant associations were identified for CAG, as shown in Table 5.

### 3.4 Associations with cumulative lifestyle scores

The CAG/BAG associations with cumulative lifestyle scores are provided in Table 5. Cumulative lifestyle scores associated negatively with BAG (*β* = −.06, 95% CI = [−0.12, −0.01], *p* = .01). However, this association did not survive corrections for multiple comparisons, as shown in Table 5. No relationship was observed between cumulative lifestyle scores and CAG (*β* = −.01, 95% CI = [−0.05, 0.02], *p* = .473, *p*_*corr*_ = .526). The results showed no difference between the BAG and CAG associations with cumulative lifestyle scores (*Z* = 0.23, *p* = .234, *p*_*corr*_ = .234), as shown in Table 6.

### 3.5 Sensitivity analyses

After the exclusion of extreme outliers (*n* of remaining sample = 532), the age‐adjusted associations between CAG and premorbid IQ (*β* = −.07, 95% CI = [−0.10, −0.03], *p* < .001, *p*_*corr*_ = .004) and education (*β* = −.06, 95% CI = [−0.09, −0.03], *p* = .001, *p*_*corr*_ = .001) were replicated. The trend between cumulative lifestyle scores and BAG was also detected (*β* = −.06, 95% CI = [−0.12, −0.01], *p* = .019, *p*_*corr*_ = .155). The relationship between *high and decreasing lifestyles* and BAG was reduced to nonsignificant (*β* = −.14, 95% CI = [−0.28, 0.00], *p* = .054, *p*_*corr*_ = .135).

Adjusting for age and other covariates (Model 2), the negative association between premorbid IQ and CAG remained significant (*β* = −.06, 95% CI = [−0.10, −0.01], *p* = .029, *p*_*corr*_ = .045), while the association between education and CAG was no longer detected (*β* = −.03, 95% CI = [−0.07, 0.02], *p* = .233, *p*_*corr*_ = .233). The trend between cumulative lifestyle scores and BAG (Model 1) was significant after further covariate adjustments (*β* = −.06, 95% CI = [−0.12, −0.01], *p* = .030, *p*_*corr*_ = .045). Premorbid IQ was more strongly associated with CAG compared to BAG (*Z* = −3.03, *p* = .234, *p*_*corr*_ = .002), while no difference was found between the BAG/CAG associations with cumulative lifestyle scores (*Z* = 1.15, *p* = .234, *p*_*corr*_ = .252).

### 3.6 Post‐hoc analyses

Given the significant association for cumulative lifestyle indices (prior to multiple comparison corrections; *p* = .029, *p*_*corr*_ = .064), we investigated whether the use of binarized indices to compute our lifestyle score may have attenuated our ability to detect the associations of interest. Specifically, a cumulative lifestyle measure based on continuous versions of the variables (where available) was examined in a series of post hoc analyses. As shown in SI Table S7, a negative association between cumulative lifestyle scores and BAG was detected in both Model 1 (*β* = −.24, 95% CI = [−0.35, −0.13], *p* = <.001, *p*_*corr*_ = <.001) and Model 2 (*β* = −.23, 95% CI = [−0.34–0.13], *p* = <.001, *p*_*corr*_ = <.001). No significant associations were found for CAG (SI Table S7, *p*'s > .05). Cumulative lifestyle scores were more strongly associated with BAG than CAG (*Z* = 2.813, *p* = .005, *p*_*corr*_ = .015), SI Table S8.

In addition, we investigated the associations between BAG/CAG and each of the lifestyle variables independently. A significant association was found between BAG and smoker status over time (i.e., individuals who reported being a “current smoker” for 3+ phases were coded as 1); Model 2 *β* = .3, 95% CI = [0.01, 0.59], *p* = .045, *p*_*corr*_ = <.045) and cumulative alcohol consumption (Model 2 *β* = .08, 95% CI = [0.03, 0.15], *p* = .003, *p*_*corr*_ = .004). None of the lifestyle variables were significantly associated with CAG (SI Table S7, *p*'s > .05). Additionally, cumulative alcohol consumption was more strongly associated with BAG than CAG (*Z* = −2.456, *p* = .014, *p*_*corr*_ = .021), whereas for smoker status over time, there was no difference in the strength of associations (*Z* = −1.566, *p* = .117, *p*_*corr*_ = .117, SI Table S8).

## 4 DISCUSSION

The present study is the first to demonstrate that by combining machine learning, MRI, and cognitive data, it is possible to quantify the concepts of brain maintenance (i.e., BAG) and cognitive maintenance (i.e., CAG) in parallel. Overall, we found no significant association between BAG and CAG. While this potentially supports the idea that there is limited correspondence between brain and cognitive maintenance in older age (Stern, 2002), the accuracy of the cognitive age prediction was relatively low, which may have limited sensitivity to detecting BAG and CAG relationships. However, the lack of association was also replicated when BAG was tested against measures of global and domain‐specific cognitive function. Moreover, premorbid IQ was related to CAG independent of BAG, in line with established literature highlighting IQ as a potential proxy for cognitive reserve (Anthony & Lin, 2018). Our results are, however, in contradiction with other studies showing consistent links between BAG and cognitive performance (e.g., Boyle et al., 2020; Cole et al., 2018; Elliott et al., 2019). This discrepancy could potentially be attributed to disparities between the cognitive tests administered, or differences in sample characteristics.

### 4.1 Brain maintenance versus cognitive maintenance: Potential utility of machine learning

While brain age prediction is frequently used as a metric of brain preservation in aging (Franke & Gaser, 2019), there is currently no established method for capturing preserved cognitive function relative to degree of brain decline in cross‐sectional studies. To date, the measurement of cognitive reserve remains a controversial topic (Jones et al., 2011; Satz, Cole, Hardy, & Rassovsky, 2011), particularly given the lack of interstudy agreement in the type or number of indices used.

Measures of cognitive reserve have traditionally consisted of either a stand‐alone measure of education or premorbid IQ, or a combination of these indices (alongside occupational complexity and engagement in leisure activities (Valenzuela & Sachdev, 2006a; Valenzuela & Sachdev, 2006b)). The most commonly used index of reserve is education, with the rationale being that education may contribute to reserve by promoting cognitive flexibility (Stern, 2002) or accumulation of neural resources (Cabeza et al., 2018). Despite evidence to suggest that education is consistently linked to a lower risk of dementia (Anstey, Ee, Eramudugolla, Jagger, & Peters, 2019), and may moderate the relationship between neuropathology and cognitive performance (Bennett et al., 2003; Rentz et al., 2010), the use of education as a “proxy” of cognitive reserve is conceptually problematic: although cognitive activity may promote cognitive reserve, such mechanisms are unlikely to represent the only pathway between education and late‐life cognition. Genetics and early life factors such as birth weight, childhood IQ, and parental education have been shown to influence brain and cognition continuously across the lifespan (Finkel, Reynolds, McArdle, & Pedersen, 2005; Lyons et al., 2009; Walhovd et al., 2016), and associations between education and cognitive function may be further mediated by socioeconomic status, risk of disease (e.g., heart disease, stroke and diabetes (Jones et al., 2011; Reed et al., 2010)), lifestyle factors, as well as individual potential for neural compensation and plasticity (Cabeza et al., 2018; Fjell et al., 2014). Educational attainment may therefore be better regarded as one of several *predictors* of cognitive maintenance in aging.

Another important consideration is that *cognitive reserve* refers to the *deviation* between observed cognitive function and what is expected based on the degree of age‐ or disease‐related brain changes (Stern, 2002). Therefore, any measure of cognitive reserve needs to take into account the disparity between observed cognitive performance and underlying brain structure. In line with this, some efforts to improve measurements of this construct have defined cognitive reserve as the residual variance in episodic memory, after adjusting for demographic variables and structural MRI abnormalities (Reed et al., 2010). This approach to characterizing cognitive reserve as model “residuals” appears promising (Reed et al., 2010; Zahodne et al., 2013). For example, the residual term has been linked to risk of conversion from mild cognitive impairment to dementia, and has been shown to modify the relationship between brain atrophy and cognitive decline (Reed et al., 2010). However, this body of evidence has focused exclusively on a single cognitive domain (memory), and considered only a handful of MRI markers of brain integrity, including global and hippocampal GM and WMHs. While the approach in the current study also utilizes model residuals, the use of machine learning provides the advantage of integrating a substantially greater number of MRI variables, as well as a large body of tests measuring performance across multiple cognitive domains. Furthermore, the age prediction models add a dimension to cross‐sectional studies by capturing deviations from normative aging trajectories.

By generating separate measures of brain and cognitive maintenance, we are able to shed light on cognitive reserve mechanisms by (a) examining the relationship between brain and cognitive maintenance, (b) identifying discrepancies between their associations with variables of interest, and (c) mutually adjusting for these variables in the same model. In this way, our approach may contribute to addressing crucial questions in the aging field, including whether lifestyle factors predominately contribute to brain maintenance or cognitive reserve in late life.

### 4.2 Favorable lifestyles are linked to higher brain maintenance

The extent to which brain maintenance and cognitive reserve form pathways between favorable lifestyles and cognitive maintenance in aging is currently unclear. In our main analyses, only a weak association was found between more favorable lifestyles between midlife and late life and brain maintenance, and the association did not statistically differ from the (insignificant) association between lifestyles and cognitive maintenance. However, post hoc analyses suggested that the method used to compute lifestyle scores (as the average number of health behaviors an individual participated in over time, ranging from 0 to 3) may have attenuated our ability to detect the associations of interest. When a cumulative lifestyle score based on continuous measures of PA and alcohol consumption was instead used, the relationship with brain maintenance appeared to strengthen and survive covariate and multiple comparison corrections. While our original method for estimating lifestyle engagement is in line with previous studies (i.e., count of unhealthy/healthy behaviors, (Lourida et al., 2019; Sabia et al., 2009) and the health behaviors were thresholded based on established guidelines and recommendations, our findings suggest that when brain and cognitive maintenance is of focus, lifestyle indices should integrate health behaviors in their continuous forms, where available, as this may improve sensitivity to the small effects often reported for lifestyle variables (Corley, Cox, & Deary, 2018). Using longitudinal assessments of BAG and CAG may also provide further information about how lifestyle affects brain and cognitive trajectories over time, as only cross‐sectional estimates of these constructs were available in the current study.

Examining how genetic variability (Lee, 2013) mediates the associations of interest is also an important “next step” for future studies, to better capture interactions between genes and environment. For example, Lourida et al. (2019)) found that favorable lifestyles were linked to reduced dementia risk, specifically in individuals with a genetic risk of dementia. IQ is also known as a heritable trait (Plomin & Deary, 2015), and a possible genetic correlation between this trait and cognitive reserve could indicate that the phenotypic relationship observed between these factors reflects common underlying genetic architecture (Lee et al., 2018). Future studies may therefore consider integrating polygenic risk scores and genetic pleiotropy to further capture the variance in CAG and BAG.

### 4.3 Strengths and limitations

The multimodal MRI and detailed cognitive battery administered to most participants allowed us to generate a large number of MRI and cognitive features to be used in the estimation of brain/cognitive age. While it is possible that measurement error introduced by cognitive tests may account for the reduced prediction accuracy of our cognitive model when compared to the MRI‐based predictions, re‐running these analyses with latent factors did not substantially modify the prediction accuracy of cognitive age. Although the brain‐age model included a larger number of input features than the cognitive model, the relative model performances were similar when 20 PCA‐transformed components were used as input for each model. This discrepancy in prediction accuracy may thus reflect that the cognitive measures were less related to chronological age compared to the MRI measures in the current sample.

As model performance has been shown to depend on sample characteristics (de Lange, Anatürk, et al., 2020), utilizing larger cohorts with an extended age range may help to improve the accuracy of cognitive age predictions. Moreover, our cognitive age model utilized the full sample to estimate normative patterns of cognitive aging with cross‐validation. A model trained exclusively on cognitively typical individuals (performance within 1 or 2 *SD*s of age and sex norms) and applied to a test set with a wider range of cognitive scores could potentially improve the sensitivity to variation in cognitive age estimates. Given that a train/test split of the current sample (*n* = 537) would lower the sample size, this approach would reduce the model performance (Walhovd et al., 2016) as well as the statistical power to detect associations with the predictors of interest. Hence, prospective studies utilizing larger cohorts with a wider age range would also be more suitable for investigating how modifying the composition of training sets influences model performance.

Due to the relatively low accuracy of the cognitive age prediction in the current study, the weak association between the BAG and CAG estimates should be interpreted with caution. However, BAG did not correlate with measures of global or domain‐specific cognitive function (SI Table S5), indicating that the lower accuracy of the cognitive age prediction could not fully explain the lack of association between the BAG and CAG estimates.

As compared to more traditional measures such as total GM volume and general scores of cognitive function, age prediction models distill a rich variety of brain characteristics and cognitive performance scores into single estimates per individual, which can be compared to normative age trajectories. However, summarizing measures across brain regions and cognitive domains cannot provide information about specialized cognitive networks potentially linked to regional brain characteristics. Combined with models that estimate regional brain aging patterns (de Lange, Anatürk, et al., 2020; Eavani et al., 2018; Kaufmann et al., 2019; Smith et al., 2020), a development of process‐specific cognitive models may provide a more detailed and accurate estimate of the relationship between brain and cognitive maintenance. Furthermore, dynamic brain processes that are not captured by structural measures may play an important role in reserve mechanisms (Franzmeier et al., 2017; Sala‐Llonch et al., 2012). Future studies including models based on functional MRI data may thus help to further elucidate the mechanisms involved in cognitive maintenance in aging.

One main strength of this study is the longitudinal and repeat data available on lifestyle indices (over a mean of 17 years), which was provided by a large and highly characterized sample. However, only smoker status, alcohol intake, and PA were included in the lifestyle indices, and a several other factors, including diet (Lourida et al., 2019) and sleep patterns (Tanaka & Shirakawa, 2004), may form part of a healthy lifestyle. A more comprehensive characterization of lifestyle trajectories could thus potentially increase their explanatory value.

Another consideration is that only 17.5% of the sample were female, reflecting sex distributions in the British civil service at the time of initial recruitment (1980s), which limits the generalizability of the reported results. Consistent with previous studies investigating the WHII imaging sub‐sample (Demnitz et al., 2017; Sexton et al., 2017), a “healthy volunteer effect” was further observed (SI Table S9), such that included individuals were significantly younger and had higher MoCA scores than excluded individuals. Participants in our study further appeared to be more educated, more physically active, less overweight, and less likely to smoke than the general population, based on nationally representative data sources (SI Table S10). However, no statistical comparisons were conducted due to substantial differences in the methods used to collect this information. As these sample characteristics are likely to limit the generalizability of our results, replication in more representative samples are required. Lastly, no causal conclusions can be drawn due to the observational nature of the study.

## 5 CONCLUSION

In conclusion, this study describes a novel method for characterizing brain and cognitive maintenance relative to normative trajectories in aging. While prospective studies are required to validate these metrics in other cohorts with different sample characteristics, the presented approach provides a method for quantifying and comparing brain and cognitive age estimates, which may be valuable for future studies seeking to investigate factors underlying maintenance and reserve mechanisms in older age.

## Supporting information


**Appendix** **S1:** Supplementary Information: Click here for additional data file.

**FIGURE S1** Brain age and cognitive age predictions compared to null distributions calculated from 1,000 permutations. Left plots: Average R^2^, RMSE, and MAE for the brain age model based on 10‐fold cross‐validations with 10 repetitions (red vertical line); mean ± standard deviation (SD) for R^2^ = 0.38 ± 0.11, RMSE = 3.86 ± 0.36, MAE = 3.11 ± 0.33. The null distributions are shown in gray; mean ± SD for R^2^ = −0.14 ± 0.04, RMSE = 5.25 ± 0.09, MAE = 4.28 ± 0.08. The number of permuted results from the null distribution that exceeded the mean from the cross‐validation was 0 (*p* = 1.00 × 10^−4^). Right plots: Average R^2^, RMSE, and MAE for the cognitive age model based on ten‐fold cross‐validations with 10 repetitions (red vertical line); mean ± SD for R^2^ = 0.09 ± 0.10, RMSE = 4.70 ± 0.45, MAE = 3.84 ± 0.39. The null distributions are shown in gray; mean ± SD for R^2^ = −0.07 ± 0.02; RMSE = 5.09 ± 0.05, MAE = 4.15 ± 0.05 (*p* = 1.00 × 10^−4^).

**FIGURE S2** The association between predicted and chronological age shown for each of the models. CI = confidence interval.

## Data Availability

The study follows MRC data sharing policies (https://www.mrc.ac.uk/research/policies-and-guidance-forresearchers/data-sharing). In accordance with these guidelines, data from the Whitehall II Imaging Sub‐study will be accessible via the Dementias Platform UK (https://portal.dementiasplatform.uk/) 430 after 2019. Data from the Whitehall II Study is available through formal request to the data sharing committee (https://www.ucl.ac.uk/iehc/research/epidemiology-public-health/research/whitehallII/data-sharing).
