# Supplementary material for: Prediction of brain age and cognitive age: Quantifying brain and cognitive maintenance in aging
Source: Hum Brain Mapp. 2020 Dec 14;42(6):1626–40. doi: 10.1002/hbm.25316 (PMC7978127; doi:10.1002/hbm.25316)
Supplement: Supplementary file 1 — Appendix S1: Supplementary Information: [file HBM-42-1626-s003.docx]

## Supplementary Information

**[Insert SI Figure 1]**

**[Insert SI Figure 2]**

SI Table 1: Average R, root mean square error (RMSE), and mean absolute error (MAE) for i) the brain age and cognitive age models (*main*) and ii) the same models when using a nested CV-approach for the hyper-parameter grid search for comparison (*nested CV*). RMSE and MAE are reported in years.

| **Model** | **R** | **RMSE** | **MAE** |
| --- | --- | --- | --- |
| Brain age (main) | 0.38 $\pm$ 0.11 | 3.87 $\pm$ 0.35 | 3.11 $\pm$ 0.31 |
| Brain age (nested CV) | 0.39 $\pm$ 0.02) | 3.87 $\pm$ 0.19) | 3.13 $\pm$ 0.17 |
| Cognitive age (main) | 0.09 $\pm$ 0.10 | 4.70 $\pm$ 0.42 | 3.83 $\pm$ 0.38 |
| Cognitive age (nested CV) | 0.10 $\pm$ 0.035 | 4.71 $\pm$ 0.26 | 3.79 $\pm$ 0.25 |

SI Table 2: Average R, root mean square error (RMSE), mean absolute error (MAE), and correlation ($r$) between predicted and chronological age for i) the brain-age model used in the main analyses (*main*) and ii) a brain age model where the WM and GM thickness features were first adjusted for scanner, relative head motion, sex, and ethnic background, and GM volume and area features were adjusted for scanner, relative head motion, sex, and ethnic background in addition to ICV, using linear models (*updated GM*). CI = confidence interval. RMSE and MAE are reported in years.

| **Model** | **R** | **RMSE** | **MAE** | $r$ **[95% CI]** | $p$ |
| --- | --- | --- | --- | --- | --- |
| Brain age (main) | 0.38 $\pm$ 0.11 | 3.87 $\pm$ 0.35 | 3.11 $\pm$ 0.31 | 0.63 [0.58, 0.68] | $<$ 0.001 |
| Brain age (updated GM) | 0.38 $\pm$ 0.11 | 3.87 $\pm$ 0.34 | 3.11 $\pm$ 0.30 | 0.63 [0.58, 0.68] | $<$ 0.001 |

SI Table 3: Correlation ($r$) between brain age gap (BAG) values estimated based on i) the brain-age model used in the main analyses and ii) a model where the WM and GM thickness features were first adjusted for scanner, relative head motion, sex, and ethnic background, and GM volume and area input features were adjusted for scanner, relative head motion, sex, and ethnic background in addition to ICV, using linear models. CI = confidence interval.

|  | $r$ | **[95% CI]** | $p$ |
| --- | --- | --- | --- |
| BAG main model vs BAG updated GM input | 0.98 | [0.98, 0.98] | $<$ 0.001 |

SI Table 4: Average R, root mean square error (RMSE), mean absolute error (MAE), and correlation ($r$) between predicted and chronological age for i) the full brain-age model (*main*), ii) a reduced brain age model using one summary measure per modality as input (total grey matter volume, average FA, and total white matter hyperintensities volume; *reduced*), and iii) an extended model including separate measures for deep and periventricular WM hyperintensities (*ext - WMH*). CI = confidence interval.. RMSE and MAE are reported in years.

| **Model** | **R** | **RMSE** | **MAE** | $r$ **[95% CI]** | $p$ |
| --- | --- | --- | --- | --- | --- |
| Brain age (main) | 0.38 $\pm$ 0.11 | 3.87 $\pm$ 0.35 | 3.11 $\pm$ 0.31 | 0.63 [0.58, 0.68] | $<$ 0.001 |
| Brain age (reduced) | 0.13 $\pm$ 0.13 | 4.59 $\pm$ 0.43 | 3.70 $\pm$ 0.37 | 0.40 [0.33, 0.47] | $<$ 0.001 |
| Brain age (ext -WMH) | 0.37 $\pm$ 0.11 | 3.88 $\pm$ 0.33 | 3.12 $\pm$ 0.30 | 0.63 [0.58, 0.68] | $<$ 0.001 |

SI Table 5: Age-adjusted associations between global and domain-specific cognition with BAG. 95% confidence intervals are indicated in square brackets.

| **Measure** | $r$ **[95% CI]** | $p$ |
| --- | --- | --- |
| Global Cognition | 0.01 [-0.07, 0.10] | 0.78 |
| Executive function | -0.08 [-0.17, 0.00] | 0.06 |
| Memory | 0.05 [-0.03, 0.14] | 0.21 |
| Processing Speed | -0.06 [-0.15, 0.02] | 0.15 |

SI Table 6: Results of latent class growth analyses to identify trajectories of healthy lifestyles. The model with best fit is highlighted in **Bold**.

| **Model** | **AIC** | **BIC** | **aBIC** | **Entropy** | **BLRT p** | **VLMR p** |
| --- | --- | --- | --- | --- | --- | --- |
| 1 class (linear) | 6289.91 | 6332.77 | 6301.03 | – | – | – |
| 1 class (quadratic) | 6289.91 | 6332.77 | 6301.03 | – | – | – |
| 2 classes (linear) | 6548.17 | 6591.03 | 6559.29 | 0.86 | $<$ 0.001 | $<$ 0.001 |
| 2 classes (quadratic) | 6522.48 | 6573.91 | 6535.82 | 0.87 | $<$ 0.001 | $<$ 0.001 |
| 3 classes (linear) | 6299.84 | 6355.56 | 6314.29 | 0.84 | $<$ 0.001 | $<$ 0.001 |
| **3 classes (quadratic)** | **6098.21** | **6166.79** | **6116.00** | **0.99** | **$<$ 0.001** | **0.04** |
| 4 classes (linear) | 6289.19 | 6357.77 | 6306.98 | 0.84 | $<$ 0.001 | 0.03 |
| 4 classes (quadratic) | 6087.72 | 6173.44 | 6109.95 | 0.98 | $<$ 0.001 | $<$ 0.001 |
| 5 classes (linear) | 6295.19 | 6376.63 | 6316.32 | 0.86 | 1.00 | 0.50 |
| 5 classes (quadratic) | 6074.78 | 6177.64 | 6101.46 | 0.87 | 0.01 | $<$ 0.001 |

SI Table 7: Associations with BAG and CAG for a cumulative lifestyle measure based on continuous measures of health behaviors, where available. This included weekly units of alcohol consumed reported at each study phase, hours of moderate-to-vigorous intensity exercise reported at each study phase and smoker status (individuals were coded as “1” for every phase they self-reported being a “current smoker”, or otherwise a “0”). Note that alcohol intake and smoker status were reverse coded and all lifestyle variables were standardized before computing the average across all three lifestyle variables (and across all phases) to create the cumulative lifestyle score. The independent associations for each variable are also reported, where physical activity levels and alcohol consumption were log-transformed to address skewed distributions, and the smoker status was coded such that a “1” represented individuals who self-reported smoking over 3 or more study phases. 95% confidence intervals are indicated in square brackets. Model 1 was adjusted for chronological age only. Associations that were significant (before multiple comparison corrections) were submitted to linear regression with additional co-variates (i.e. model 2). Model 2 was adjusted for age, sex, education, ethnicity, BMI and mutual adjustments between BAG and CAG. When individual health behaviors were examined in relation to BAG and CAG, we also adjusted for participation in the other health behaviors of interest. Confidence intervals are indicated in square brackets. *P*-values are provided before and after FDR-correction.

|  |  | **Model 1** |  |  | **Model 2** |  |  |
| --- | --- | --- | --- | --- | --- | --- | --- |
| **DV** | **IV** | $\beta$ **[95% CI]** | $p$ | $p_{corr}$ | $\beta$ **[95% CI]** | $p$ | $p_{corr}$ |
| CAG | Cumulative lifestyle | -0.06 [-0.13, 0.01] | 0.08 | 0.16 | – | – |  |
|  | Alcohol consumption* | -0.01 [-0.04, 0.03] | 0.589 | 0.664 | – | – |  |
|  | Physical activity* | -0.03 [-0.06, 0.01] | 0.134 | 0.214 | – | – |  |
|  | Smoker status* | 0.04 [-0.14, 0.22] | 0.664 | 0.664 | – | – |  |
| BAG | Cumulative lifestyle | -0.24 [-0.35, -0.13] | $<$ 0.001 | $<$ 0.001 | -0.23 [-0.34, -0.13] | $<$ 0.001 | $<$ 0.001 |
|  | Alcohol consumption* | 0.09 [0.04, 0.15] | 0.003 | 0.005 | 0.08 [ 0.03, 0.15] | 0.003 | 0.004 |
|  | Physical activity* | -0.03 [ -0.08, 0.03] | 0.297 | 0.396 | – | – |  |
|  | Smoker status* | 0.39 [ 0.1, 0.68] | 0.008 | 0.025 | 0.3 [0.01, 0.59] | 0.045 | 0.045 |

**Abbreviations:-** BAG = Brain Age Gap; CAG = Cognitive Age Gap; DV = Dependent Variable; IV = Independent Variable. * Cumulative measure between P5 - OX.

SI Table 8: Difference between the BAG and CAG associations with cumulative lifestyle scores, cumulative alcohol consumption and smoker status over time (adjusting for all co-variates). For the calculation used to compare the difference between associations, see Eq. 1 (*Methods and Materials*) Confidence intervals are indicated in square brackets. *P*-values are provided before and after FDR-correction.

| **Variable** | $\beta_{CAG}$ | $\beta_{BAG}$ | $Z$ | $p$ | $p_{corr}$ |
| --- | --- | --- | --- | --- | --- |
| Cumulative lifestyle | -0.05 [-0.12, 0.02] | -0.23 [-0.34, -0.13] | 2.813 | $0.005$ | $0.015$ |
| Alcohol consumption | 0.00 [-0.04, 0.04] | 0.09 [ 0.03, 0.15] | -2.456 | $0.014$ | $0.021$ |
| Smoker status | 0.02 [-0.16, 0.21] | 0.30 [0.01, 0.59] | -1.566 | $0.117$ | $0.117$ |

SI Table 9: Comparison of included and excluded participants.

|  | **Included** | **Excluded** | **Test-statistic** | **p-value** |
| --- | --- | --- | --- | --- |
| Age | 69.61 $\pm$ 4.99 | 70.39 $\pm$ 5.52 | t = -1.94* | 0.05 |
| MoCA | 27.31 $\pm$ 2.16 | 26.82 $\pm$ 2.57 | t = 2.66* | 0.01 |
| N. of females (%) | 94 (17.5%) | 58 (22.05%) | $\chi^{2}$ = 2.09 | 0.15 |
| Education | 16.75 $\pm$ 4.44 | 16.62 $\pm$ 4.58 | t = 0.38 | 0.71 |

**Abbreviations:-** MoCA = Montreal Cognitive Assessment. NB Mean $\pm$ Standard Deviation. ^*^Levene’s test was significant (p $<$ 0.05), therefore Welch’s t-test is reported.

SI Table 10: Comparison of demographic and lifestyle factors in the Whitehall Imaging sample to nationally representative samples. As a way of aligning the census data to the Whitehall sample, we report summary statistics around the time of recruitment for the MRI sub-study (2012), for age ranges that overlap with our sample (e.g., 65-74).

| **Variables** | **Whitehall Imaging** | **Nationally representative** |
| --- | --- | --- |
|  | **sample (n = 537)** | **sample** |
| **Education**, years | 16.75 $\pm$ 4.44 | 12a |
| **Ethnicity**, White (%) | 508 (94.6%) | 94.7% for 60 - 65 year old^sb^ |
|  |  | 95.5% for 65+ year olds^b^ |
| **BMI**, $kg/m^{2}$ | 26.02 $\pm$ 3.69 for men | 28.5 for men^c^ |
|  | 25.92 $\pm$ 4.9 for women | 28.1 for women^c^ |
| **Physical activity**, 2.5 hours per week of MVPA | 333 (75.2%) for men | 58% for men^d^ |
|  | 51 (54.3%) for women | 52% for women^d^ |
| **Smoker status**, current smokers (%) | 13 (2.9%) for men | 12% for men^e^ |
|  | 2 (2.1%) for women | 12% for women^e^ |
| **Alcohol consumption**, units per week | 16.2 $\pm$ 14.9 for men | 16.9 $\pm$ 0.8 in men^f^ |
|  | 8.1 $\pm$ 9 for women | 9.3 $\pm$ 1 in women^f^ |

Abbreviations:- MVPA = moderate-to-vigorous physical activity. Note:- Mean $\pm$ SD reported, where possible. All variables reported for the Whitehall cohort were collected at time of scan (Phase OX, 2012 - 2016).

^a^ Mean years of Education for the United Kingdom in 2012, UNESCO Institute for Statistics (2019) [online]. Available at: http://hdr.undp.org/en/indicators/103006#a [Accessed 21st October 2020].

^b^ Statistics for England and Wales. Office for National Statistics; National Records of Scotland; Northern Ireland Statistics and Research Agency (2017): 2011 Census aggregate data. UK Data Service (Edition: February 2017). DOI: urlhttp://dx.doi.org/10.5257/census/aggregate-2011-2

^c^ Calculated for men and women aged between 65-74, note that standard deviations were not available. Health Survey England (HSE) 2012: Adult anthropometric measures, overweight and obesity [online] Available at: https://files.digital.nhs.uk/publicationimport/pub13xxx/pub13218/hse2012-ch10-adult-bmi.pdf [Accessed 21st October 2020].

^d^ For men and women aged between 65-74. Health Survey England (HSE) 2012: Physical activity in adults [online] Available at: https://files.digital.nhs.uk/publicationimport/pub13xxx/pub13218/hse2012-ch2-phys-act-adults.pdf [Accessed 21st October 2020].

^e^ Prevalence of cigarette smoking in men and women aged 60+ (2010). Health and Social Care Information Centre (HSCIC), Lifestyles Statistics. Statistics on Smoking: England, 2012 [online]. Available at: https://files.digital.nhs.uk/publicationimport/pub07xxx/pub07019/smok-eng-2012-rep.pdf

^f^For men and women aged between 65-74. Health Survey England (HSE) 2012: Alcohol Consumption. [online] Available at: Available at: https://files.digital.nhs.uk/publicationimport/pub13xxx/pub13218/hse2012-ch6-alc-cons.pdf
