# Supplementary figures and images for: Prediction of brain age and cognitive age: Quantifying brain and cognitive maintenance in aging
Source: Hum Brain Mapp. 2020 Dec 14;42(6):1626–40. doi: 10.1002/hbm.25316 (PMC7978127; doi:10.1002/hbm.25316)

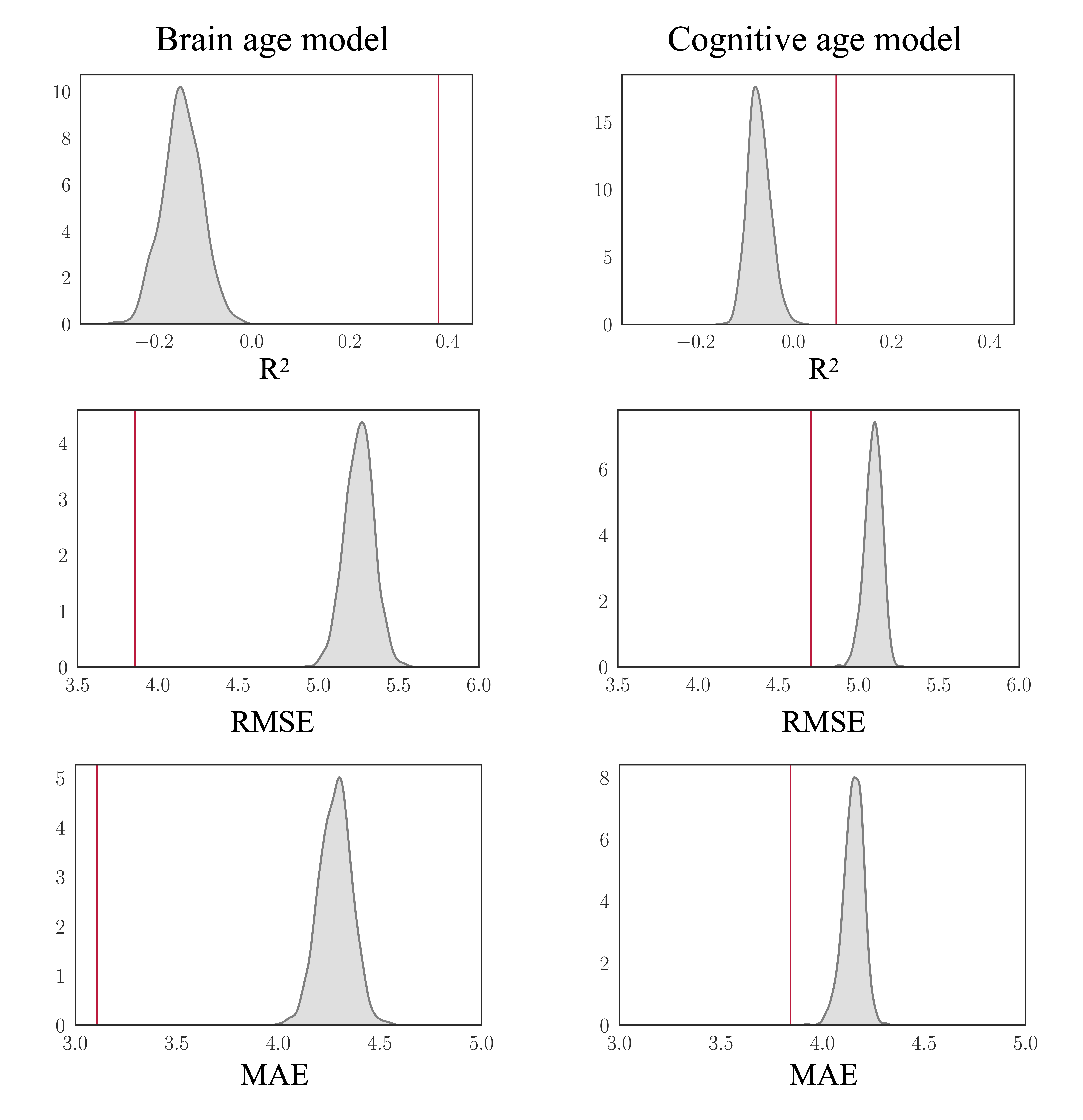

Supplement: Supplementary file 2 — FIGURE S1 Brain age and cognitive age predictions compared to null distributions calculated from 1,000 permutations. Left plots: Average R2, RMSE, and MAE for the brain age model based on 10‐fold cross‐validations with 10 repetitions (red vertical line); mean ± standard deviation (SD) for R2 = 0.38 ± 0.11, RMSE = 3.86 ± 0.36, MAE = 3.11 ± 0.33. The null distributions are shown in gray; mean ± SD for R2 = −0.14 ± 0.04, RMSE = 5.25 ± 0.09, MAE = 4.28 ± 0.08. The number of permuted results from the null distribution that exceeded the mean from the cross‐validation was 0 (p = 1.00 × 10^−4^). Right plots: Average R2, RMSE, and MAE for the cognitive age model based on ten‐fold cross‐validations with 10 repetitions (red vertical line); mean ± SD for R2 = 0.09 ± 0.10, RMSE = 4.70 ± 0.45, MAE = 3.84 ± 0.39. The null distributions are shown in gray; mean ± SD for R2 = −0.07 ± 0.02; RMSE = 5.09 ± 0.05, MAE = 4.15 ± 0.05 (p = 1.00 × 10^−4^). [file HBM-42-1626-s001.tiff]

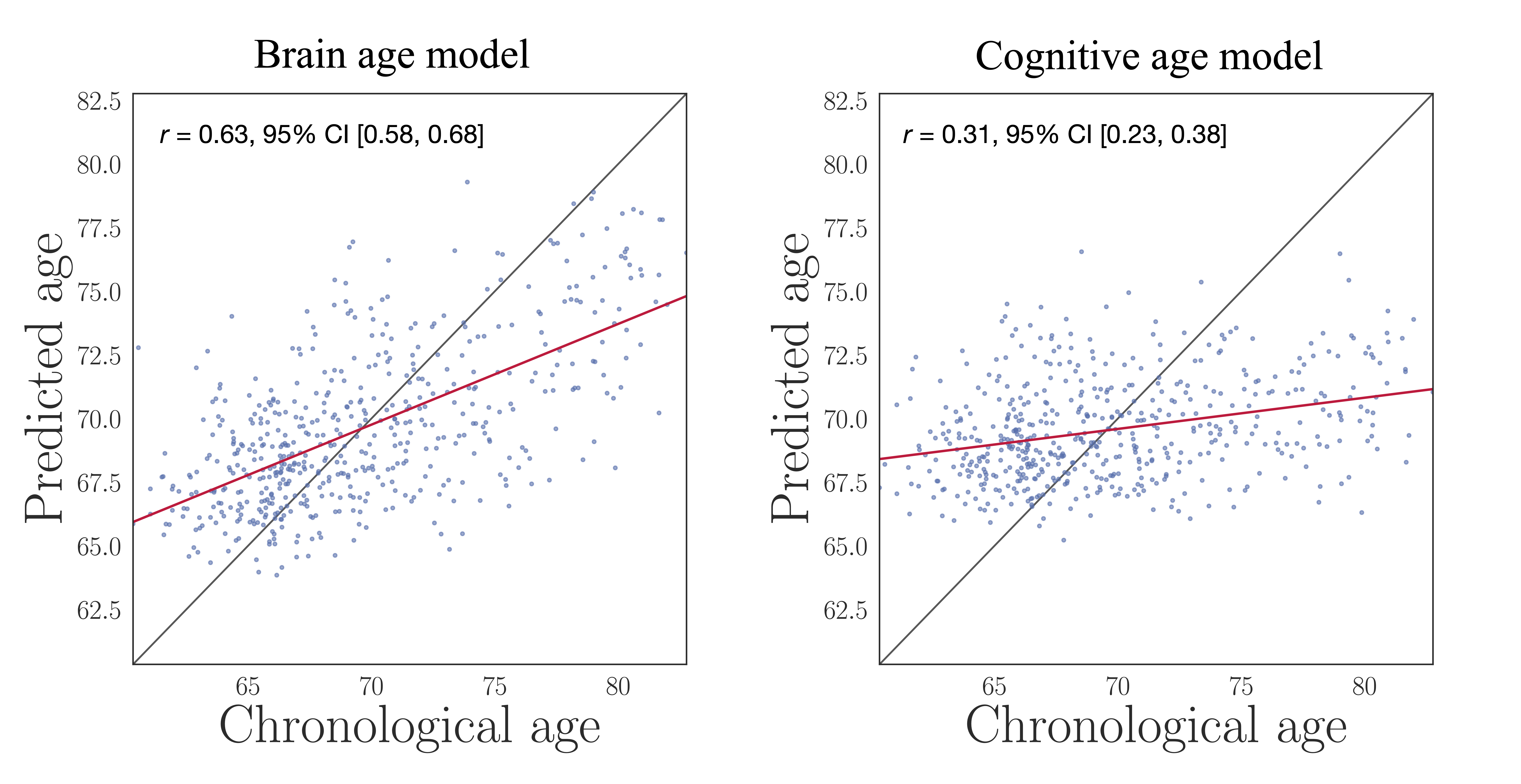

Supplement: Supplementary file 3 — FIGURE S2 The association between predicted and chronological age shown for each of the models. CI = confidence interval. [file HBM-42-1626-s002.tiff]
